# Verapamil increases the sensitivity of primary human colorectal carcinoma tissue to vincristine.

**DOI:** 10.1038/bjc.1986.19

**Published:** 1986-01

**Authors:** P. Ince, D. R. Appleton, K. J. Finney, J. P. Sunter, A. J. Watson


*Br. J. Cancer* (1986), **53**, 137–139

### Short Communication

### Verapamil increases the sensitivity of primary human colorectal carcinoma tissue to vincristine

P. Ince[1], D.R. Appleton[2], K.J. Finney[1], J.P. Sunter[1] & A.J. Watson[1]

*Departments of [1]Pathology and [2]Medical Statistics, University of Newcastle upon Tyne, Royal Victoria Infirmary, Queen Victoria Road, Newcastle upon Tyne NE1 4LP, UK.*

The inherent resistance of human solid tumours to cancer chemotherapy is a major problem in medical oncology. Experimentally, in cell lines, the resistance phenomena studied are often induced by a process analogous to the induction of antibiotic resistance in bacteria. The acquired mechanisms by which resistant mutants differ from parent cells are studied and less attention paid to the differences between the innate resistance of a tumour and the normal cell population from which it arose. There is an urgent need to relate the results obtained in these systems to human tumour tissue.

Verapamil, a calcium transport antagonist widely used in cardiological medicine, has been shown to be a potent modifier of vincristine resistance in several cell lines (Tsuruo *et al.*, 1981 & 1983). Clinical trials using verapamil as a modifier of vindesine therapy in advanced human malignancy are in progress (Cantwell *et al.*, 1985). In the present study we have examined the effects of verapamil on the sensitivity of primary human colonic carcinomata to vincristine.

In the formal stathmokinetic experiment a dose of vincristine is administered, large relative to clinical therapy (i.e. approximately 15 times greater for human colon cancer), to arrest all dividing cells in metaphase. The resulting "arrested metaphase" figures are easily recognised in histological preparations, and their rate of accumulation is used to calculate the cell birth rate. Using this technique we have previously demonstrated that a six times greater dose of vincristine is required to achieve a maximum rate of metaphase accumulation in human colonic tumour tissue than that required for normal colonic mucosa (Pritchett *et al.*, 1982). We have recently developed a method of measuring directly the degree of escape from metaphase arrest by counting the proportion of mitotic figures showing anaphase or telophase configuration (Ince *et al.*, 1985). The resulting Post-Metaphase Index (PMI) can be used in a "dose-response" format at a range of vincristine doses extending down to the human therapeutic range (i.e. 200–300 nmol plasma levels). This is the technique we have used in assessing changes in vincristine resistance in the present study.

Eleven human colorectal carcinomata were studied. All were left-sided lesions treated by abdominoperineal resection, anterior resection, or left hemicolectomy. The specimens were collected and cleaned in theatre, and transported to the laboratory in ice-cold medium. Apparently viable areas of tumour tissue were identified, from which explants, measuring $\sim 2\,mm^3$, were prepared. The explants were placed on millipore filters in 60 mm plastic petri dishes and were cultured in Weymouth's MB752/1 medium supplemented with 10% foetal calf serum, hydrocortisone, vitamin C, and ferrous sulphate. The dishes were maintained in a controlled atmosphere chamber with a gas phase of 95% $O_2$, 5% $CO_2$, using a rocking culture technique (Senior *et al.*, 1984).

Altogether 120 explants per tumour were used with four explants to each dish. After 16 h incubation the dishes were divided into three groups and the medium was changed to include verapamil at one of three doses *viz.* 0.0 $\mu$mol, 6.6 $\mu$mol, 13.2 $\mu$mol. After a further 2 h the media were again changed to include verapamil at the same dose and vincristine at one of five doses, *viz.* 270 nmol, 540 nmol, 1080 nmol, 2160 nmol, 4320 nmol. After 2 h incubation with vincristine the tissues were fixed in formalin and routinely processed to paraffin wax. Histological sections were prepared at 4 $\mu$m and stained with haematoxylin and eosin.

The PMI was derived by evaluating a total of at least 50 tumour cell mitotic figures in step sections of each explant. Multiples of a whole section of each explant were counted including that in which the 50th mitosis was identified. The proportion of post-metaphase mitotic figures was recorded. At each combination of verapamil and vincristine doses the data from the several explants were pooled to provide a mean PMI. Out of eleven tumours six were rejected prior to counting because

Correspondence: P. Ince.
Received 17 July 1985; and in revised form 12 September 1985.

© The Macmillan Press Ltd., 1986

of unsuccessful culture; failure was due to bacterial contamination, tumour necrosis, or the fact that some tumours were scirrhous with much stroma and little epithelial tissue. From the five successful tumour cultures 45% of explants were unsuitable for counting, owing to excessive necrosis, or the presence of only minimal epithelial tumour tissue. Some of the "unsuitable" tumour explants comprised normal colonic mucosa only.

The five tumours which were cultured successfully were all graded histologically as moderately differentiated adenocarcinomata. Two were from the sigmoid colon and three from the rectum. Dukes' staging was as follows: stage A – 1 tumour; stage B – 2 tumours; stage C – 1 tumour; stage D – 1 tumour.

The pooled data from all five tumours are shown in Table I. Figure 1 is a three-dimensional plot of the relationship between the PMI (%) and vincristine dose at the three verapamil doses used. At all three doses there is an increase in metaphase escape with decreasing dose of vincristine, and this

**Table I** Mean Post-Metaphase Index with standard errors.

| *vincristine dose (nmol)* | *verapamil dose (μmol)* | | |
|---|---|---|---|
| | *0.0* | *6.6* | *13.2* |
| 270 | 4.6±1.3 | 2.9±0.7 | 3.3±1.5 |
| 540 | 3.4±1.4 | 2.6±0.6 | 2.0±0.6 |
| 1080 | 2.4±1.4 | 0.5±0.3 | 1.0±0.6 |
| 2160 | 0.5±0.2 | 0.3±0.3 | 0.2±0.1 |
| 4320 | 0.0 | 0.1±0.1 | 0.3±0.3 |

corresponds with our previous observations on this type of data. Verapamil at doses of both 6.6 μmol, and 13.2 μmol, comparable with therapeutic plasma levels up to 10 μmol (Cantwell *et al.*, 1985) causes a similar and statistically significant degree of enhancement of the effect of vincristine. No simple function of the PMI is linearly related to dose or log dose of vincristine. Analysis using the GLIM programme (Baker & Nelder, 1978) and a logistic transformation of the PMI shows that either dose of verapamil reduces the PMI by a factor of 0.61 (95% confidence limits 0.47 to 0.78, $P<0.001$) independently of the dose of vincristine. A satisfactory fit ($\chi^2_9=10.91$) is found by fitting the model;

Logit PMI = −6.34 − 0.50 if verapamil
(s.e. 0.13 $P<0.001$)
+0.00 if 4320 nmol of vincristine
1.01 if 2160 nmol of vincristine
1.27 if 1080 nmol of vincristine
3.05 if 540 nmol of vincristine
3.35 if 270 nmol of vincristine

These results provide direct experimental evidence of the efficacy of pharmacological modification of primary solid human tumour resistance to vincristine.

Acquired tumour cell resistance to vincristine is frequently associated with cross-resistance to anticancer drugs of differing modes of action, notably adriamycin. This has been termed the pleiotropic multidrug-resistance phenotype, and in animal tumour-cell lines appears to be related to the presence of, and phosphorylation status of, a cell

**Figure 1** Three dimensional plot of the effects of vincristine and verapamil on the postmetaphase index.

surface glycoprotein designated P180 (Ling *et al.*, 1983; Garman *et al.*, 1983). A wide range of pharmacological modifiers of both vincristine and adriamycin resistance have been described in a variety of cell lines of human and animal origin displaying either acquired or inherent drug resistance. The pharmacologically induced increase in tumour-cell sensitivity is accompanied, and possibly caused, by increased intracytoplasmic accumulation of the anticancer drug (Tsuruo *et al.*, 1982). Thus the postulated mechanism of resistance is the existence of a drug elimination pathway in the plasma membrane which allows cancer cells to minimise the intracellular concentration of the drug. Verapamil is thought to inhibit this pathway. However, other possible resistance mechanisms, such as increased intracytoplasmic drug binding may operate (Beck *et al.*, 1983), and the issue remains to be resolved. Currently we are using our organ culture system to investigate the underlying biochemical basis of vincristine resistance in primary colonic cancer. This is of crucial importance in the longer term development of more specific and potent modifiers for use in clinical therapy. Current experimental strategies comprise empirical selection of potential modifiers of vincristine resistance, and battery testing against a range of cell lines of varying drug resistance profile. This approach has demonstrated that different agents vary in their ability to act as modifiers depending upon the particular combination of cytotoxic drug and cell line tested (Ramu *et al.*, 1984). It is clear from our results that there is evidence *in vitro* that verapamil has a modifying effect on human primary colonic cancer cells. This may be of some clinical usefulness in the future, but verapamil will not necessarily prove to be the most effective enhancer of vincristine potency.

The verapamil used was kindly donated by Abbott Laboratories, Queenborough, Kent. We are grateful to Kathryn Elliott for the preparation of histological sections and to Brenda Kennedy who typed the manuscript. This study was supported by a grant from the North of England Cancer Research Campaign.

### References

BAKER, R.J. & NELDER, J.A. (1978). The GLIM System (Release 3) Manual. Numerical Algorithms Group for the Royal Statistical Society.

BECK, W.T., CITAIN, M.C. & LEFKO, J.L. (1983). Energy-dependent reduced drug binding as a mechanism of vinca alkaloid resistance in human leukemic lymphoblasts. *Molecular Pharmacol.*, **24**, 485.

CANTWELL, B.M., BAUMAH, P. & HARRIS, A.L. (1985). Phase I and II study of oral verapamil (VRP) and intravenous vindesine (VDN). *Proc. Am. Soc. Clin. Oncol*, **21**, 42.

GARMAN, D., ALBERS, L. & CENTER, M.S. (1983). Identification and characterisation of a plasma membrane phosphoprotein which is present in Chinese hamster lung cells resistant to adriamycin. *Biochem. Pharmacol.*, **32**, 3633.

INCE, P., FINNEY, K.J., SUNTER, J.P., APPLETON, D.R. & WATSON, A.J. (1985). Demonstration of vincristine resistance in primary intestinal neoplasms in the rat by the "Post-Metaphase Index". *Br. J. Cancer*, **52**, 599.

LING, V., KARTNER, N., SUDO, T., SIMINOVITCH, L. & RIORDAN, J.R. (1983). Multidrug-resistance phenotype in Chinese hamster ovary cells. *Cancer Treat. Rep.*, **67**, 869.

PRITCHETT, C.J., SENIOR, P.V., SUNTER, J.P., WATSON, A.J., APPLETON, D.R. & WILSON, R.G. (1982). Human colorectal tumours in short term organ culture. *Cell Tissue Kinet.*, **15**, 555.

RAMU, A., SPANIER, R., RAHAMIMOFF, H. & FUKS, Z. (1984). Restoration of doxorubicin responsiveness in doxorubicin resistant P388 murine leukaemia cells. *Br. J. Cancer*, **50**, 501.

SENIOR, P.V., SUNTER, J.P., APPLETON, D.R. & WATSON, A.J. (1984). Morphological studies on the long term organ culture of colonic mucosa from normal and dimethylhydrazine treated rats. *Br. J. Cancer*, **49**, 281.

TSURUO, T., IIDA, H., TSUKAGOSHI, S. & SAKURAI, Y. (1981). Overcoming of vincristine resistance in P388 leukemia *in vivo* and *in vitro* through enhanced cytotoxicity of vincristine and vinblastine by verapamil. *Cancer Res.*, **41**, 1967.

TSURUO, T., IIDA, H., TSUKAGOSHI, S. & SAKURAI, Y. (1982). Increased accumulation of vincristine and adriamycin in drug-resistant P388 tumor cells following incubation with calcium antagonists and calmodulin inhibitors. *Cancer Res.*, **42**, 4730.

TSURUO, T., IIDA, H., NAGANUMA, K., TSUKAGOSHI, S. & SAKURAI, Y. (1983). Promotion by verapamil of vincristine responsiveness in tumor cell lines inherently resistant to the drug. *Cancer Res.*, **43**, 808.


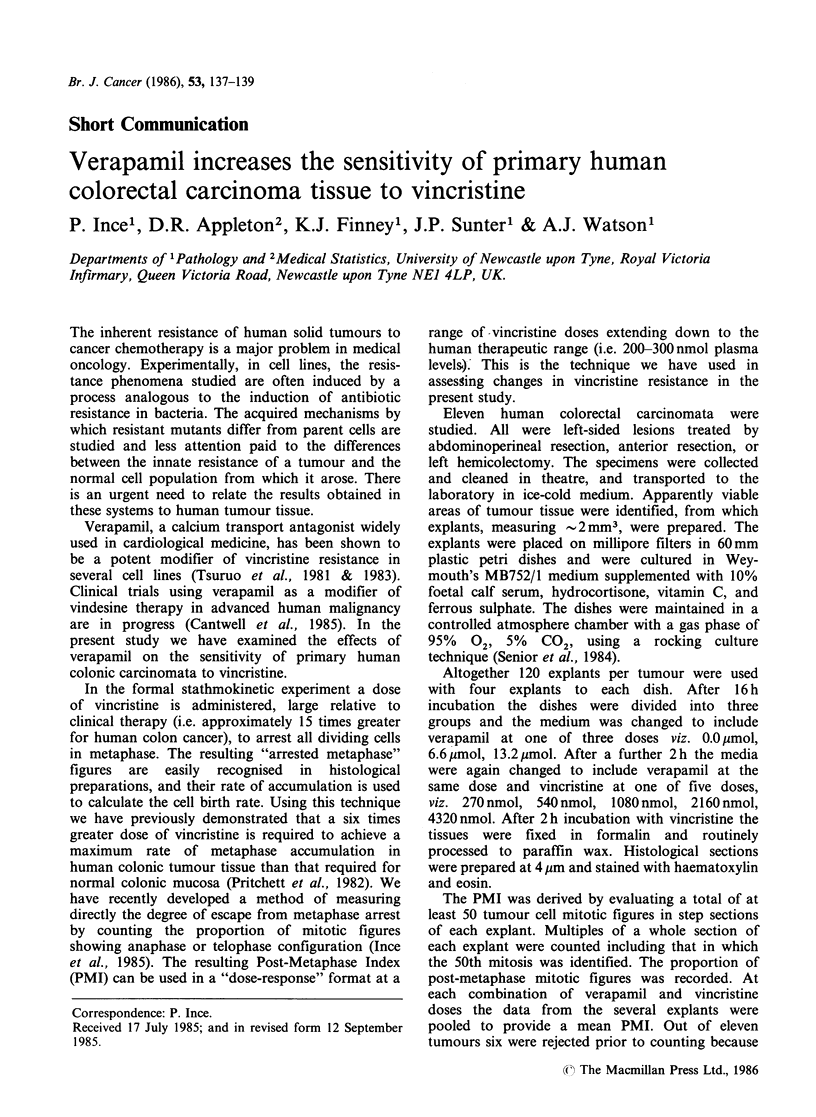

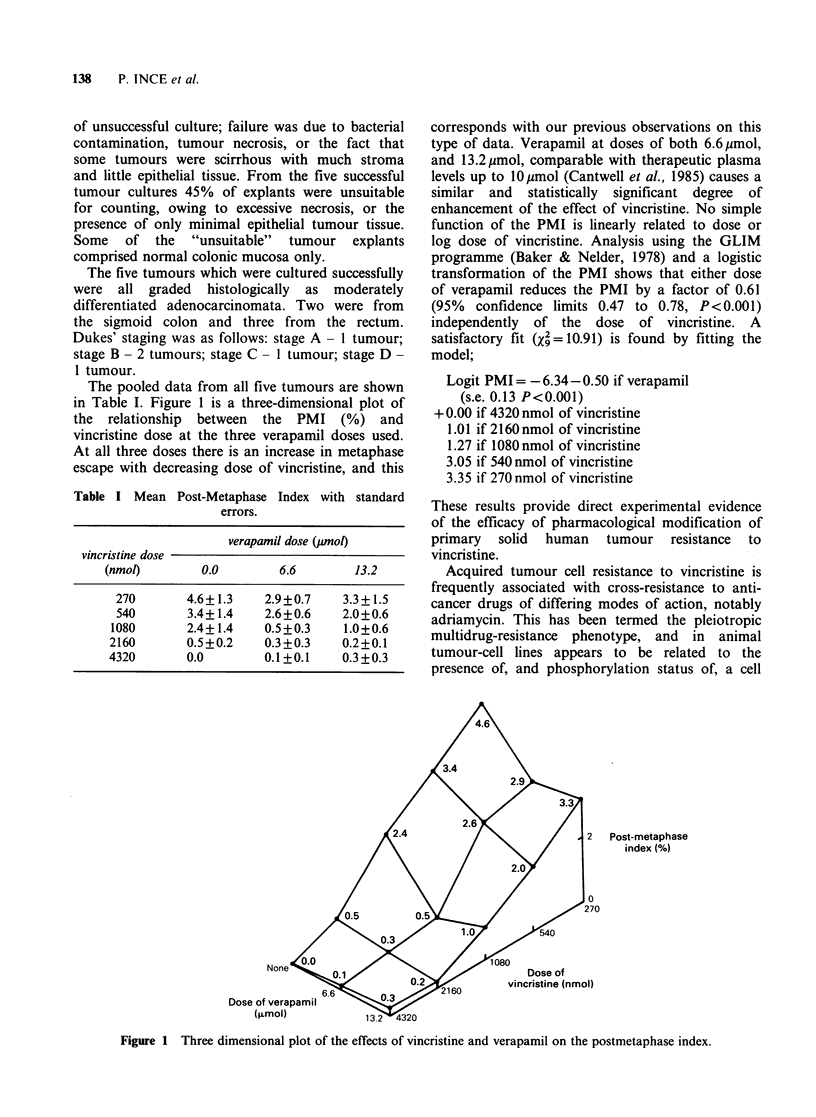

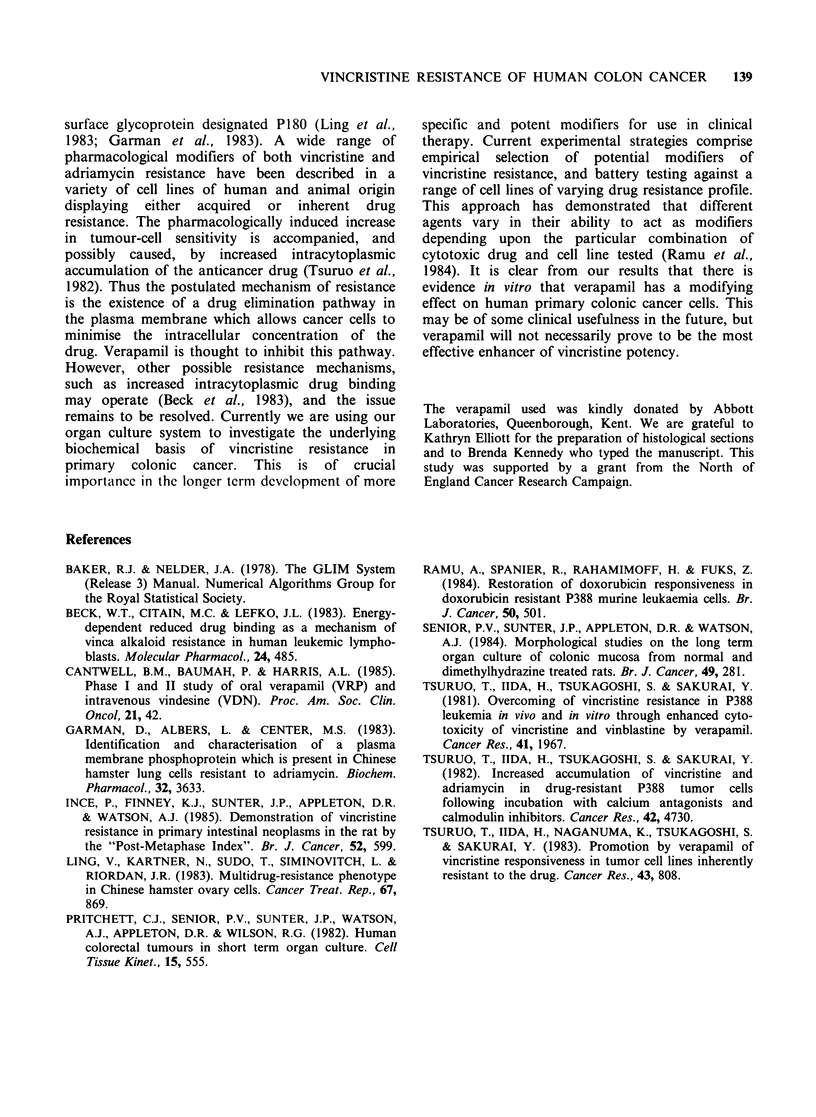


## References

[OCR_00268] Beck W. T., Cirtain M. C., Lefko J. L. (1983). Energy-dependent reduced drug binding as a mechanism of Vinca alkaloid resistance in human leukemic lymphoblasts.. Mol Pharmacol.

[OCR_00280] Garman D., Albers L., Center M. S. (1983). Identification and characterization of a plasma membrane phosphoprotein which is present in Chinese hamster lung cells resistant to adriamycin.. Biochem Pharmacol.

[OCR_00287] Ince P., Finney K. J., Appleton D. R., Sunter J. P., Watson A. J. (1985). Demonstration of vincristine resistance in primary intestinal neoplasms in the rat by the 'post-metaphase index'.. Br J Cancer.

[OCR_00292] Ling V., Kartner N., Sudo T., Siminovitch L., Riordan J. R. (1983). Multidrug-resistance phenotype in Chinese hamster ovary cells.. Cancer Treat Rep.

[OCR_00298] Pritchett C. J., Senior P. V., Sunter J. P., Watson A. J., Appleton D. R., Wilson R. G. (1982). Human colorectal tumours in short-term organ culture. A stathmokinetic study.. Cell Tissue Kinet.

[OCR_00304] Ramu A., Spanier R., Rahamimoff H., Fuks Z. (1984). Restoration of doxorubicin responsiveness in doxorubicin-resistant P388 murine leukaemia cells.. Br J Cancer.

[OCR_00310] Senior P. V., Sunter J. P., Appleton D. R., Watson A. J. (1984). Morphological studies on the long-term organ culture of colonic mucosa from normal and dimethylhydrazine treated rats.. Br J Cancer.

[OCR_00330] Tsuruo T., Iida H., Naganuma K., Tsukagoshi S., Sakurai Y. (1983). Promotion by verapamil of vincristine responsiveness in tumor cell lines inherently resistant to the drug.. Cancer Res.

[OCR_00323] Tsuruo T., Iida H., Tsukagoshi S., Sakurai Y. (1982). Increased accumulation of vincristine and adriamycin in drug-resistant P388 tumor cells following incubation with calcium antagonists and calmodulin inhibitors.. Cancer Res.

[OCR_00316] Tsuruo T., Iida H., Tsukagoshi S., Sakurai Y. (1981). Overcoming of vincristine resistance in P388 leukemia in vivo and in vitro through enhanced cytotoxicity of vincristine and vinblastine by verapamil.. Cancer Res.

